# Supplementary figures and images for: Two oxytocin analogs, N-(p-fluorobenzyl) glycine and N-(3-hydroxypropyl) glycine, induce uterine contractions ex vivo in ways that differ from that of oxytocin
Source: PLoS One. 2023 Feb 9;18(2):e0281363. doi: 10.1371/journal.pone.0281363 (PMC9910740; doi:10.1371/journal.pone.0281363)

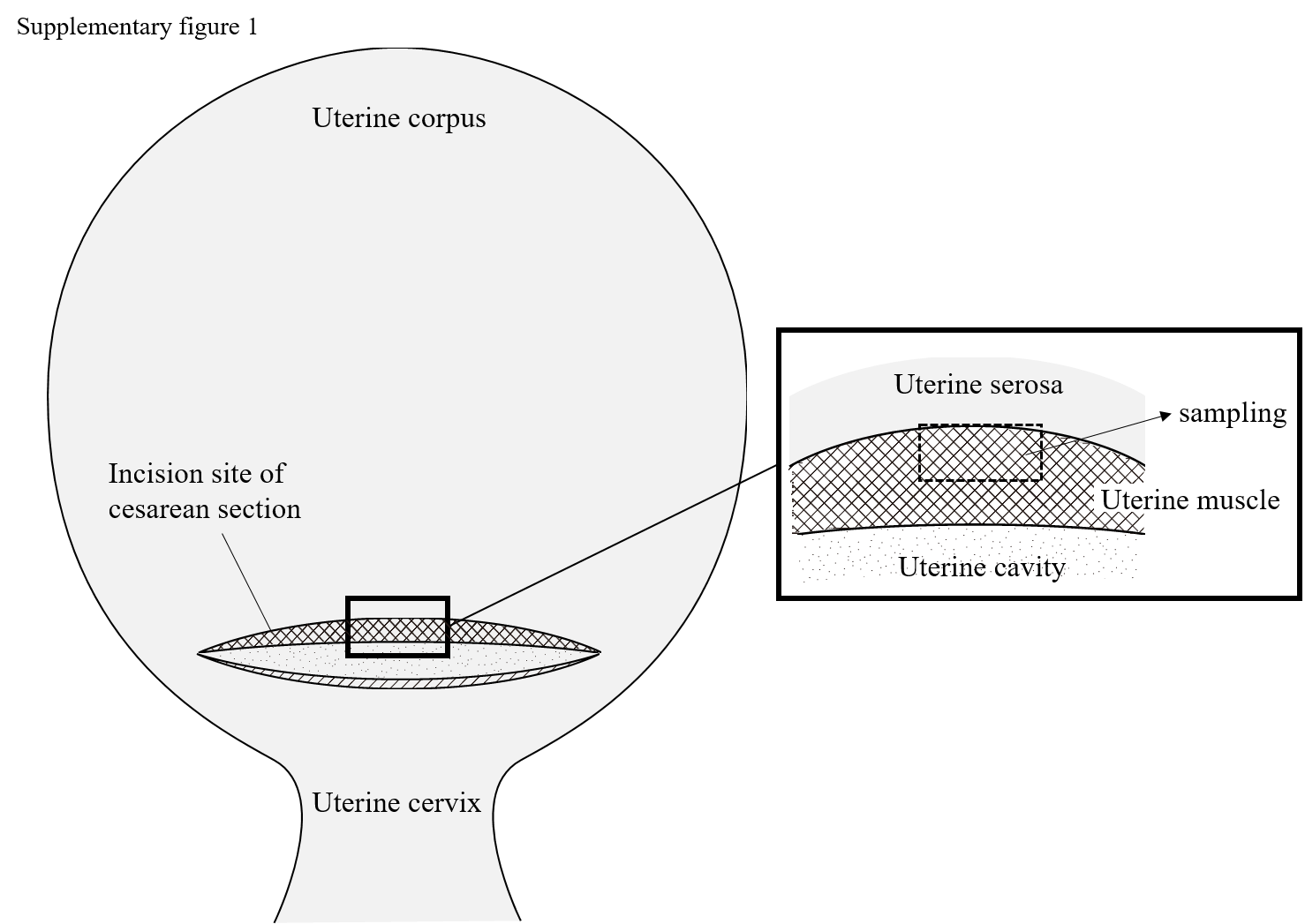

Supplement: S1 Fig — (TIF) [file pone.0281363.s002.tif]

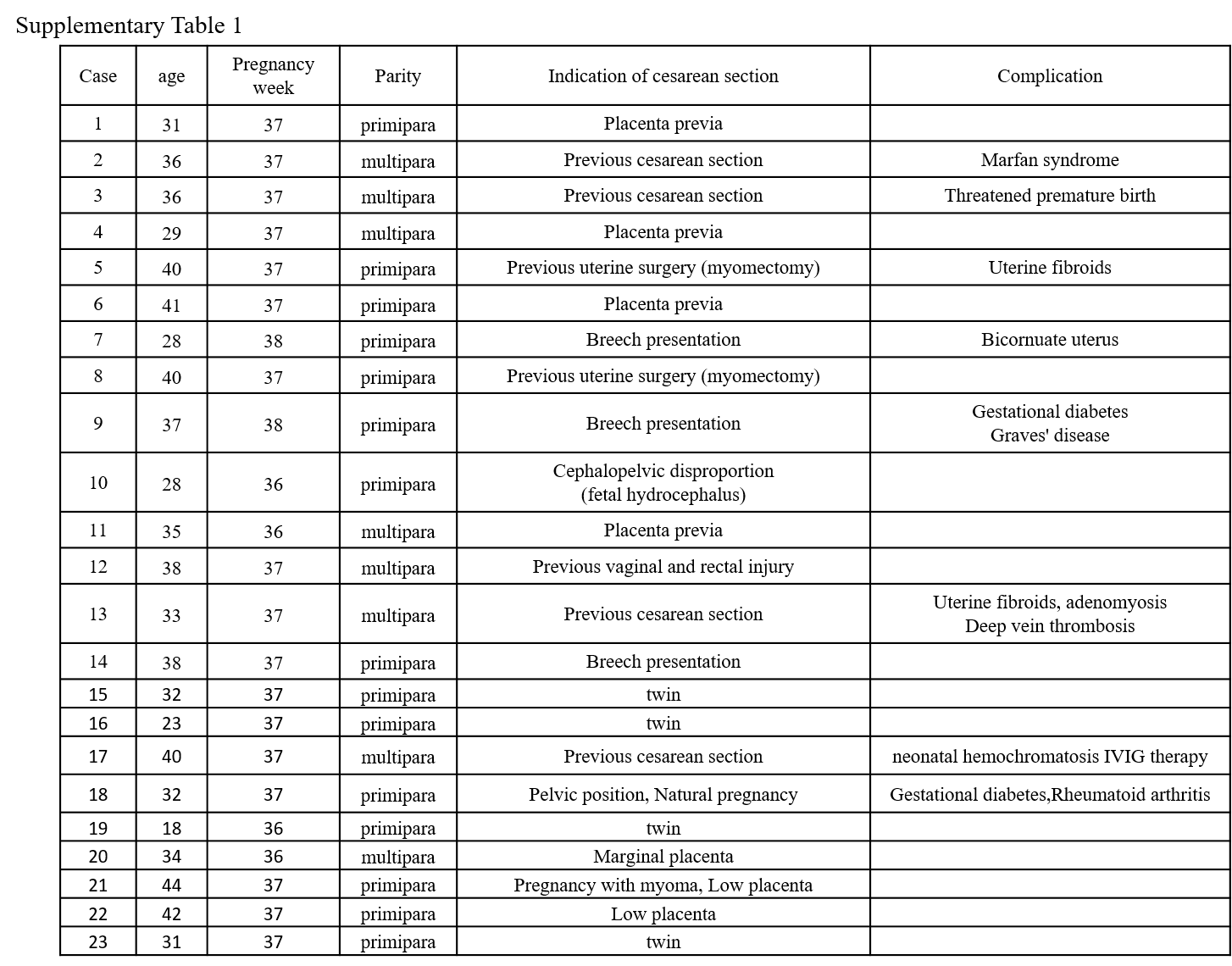

Supplement: S1 Table — (TIF) [file pone.0281363.s003.tif]
