# Supplementary material for: Two oxytocin analogs, N-(p-fluorobenzyl) glycine and N-(3-hydroxypropyl) glycine, induce uterine contractions ex vivo in ways that differ from that of oxytocin
Source: PLoS One. 2023 Feb 9;18(2):e0281363. doi: 10.1371/journal.pone.0281363 (PMC9910740; doi:10.1371/journal.pone.0281363)
Supplement: Attachment — Submitted filename: Review response.docx [file pone.0281363.s004.docx]

**Comment:** Reviewer #1: Thank you for this great work. It seems to me that it should be made clear why virgin mouse uteri and muscle fibers from pregnant women were chosen. Why did you not make a comparison between uteri of virgin mice and uteri of mice that were already pregnant?

**Response**: Thank you very much for reasonable comments and questions.

You right about necessity of comparison of virgin vs pregnant uterine tissue, we address our discrepancy for HPOT, and use of pregnant uterine tissue can be useful in this context. However, it is also important to obtain the same tissue region, because the uterus is composed of heterogeneous muscles. Usage of the virgin mouse uterus is to obtain from the same region, in which similar muscle composition is expected and it is more complicated for pregnant mice. So, for this step we limited only for virgin mice for better precision. To address this issue, we modified paragraph in discussion as

“The reason for this discrepancy is unclear. This may be because of species-specific differences and differences between non-pregnant and pregnant tissues. Therefore, the effects of HPOT have to be assessed in the pregnant uterus or uterine muscles during parturition in mouse mothers. In the current research, we limited our study only by the inclusion of virgin mice in order to obtain exactly the same tissue region for comparison, where similar muscle composition is expected.”

**Comment:** Why didn't they collect uterine muscle fibers from women who had never been pregnant?

**Response**: We considered human pregnant uteri to be the most appropriate for evaluating the effects of OT, carbetocin, FBOT, and HPOT treatments. For this reason, we received approval from the Ethics Committee of Kanazawa University, and obtained consent from pregnant women undergoing caesarean section to provide specimens. Because in that case caesarean section was appropriate procedure for patients, it’s possible to obtain approval. Collection of uterine tissue from healthy non-pregnant woman will be ethically unjustified.

**Comment:** Furthermore, it is not specified on the women from whom uterine muscle fibers were taken whether it was a first or second caesarean section.

**Response**: Thanks for notice. We add next line to the methods description:

“There were 3 secondary and 20 primary cesarean sections out of 23.”

**Comment:** Indeed, during a second caesarean section, the risk is to take fibrous tissue which would create a bias during the measured contractions.

**Response**: For the second caesarean section, we performed a caesarean section with an incision cranial to the uterine incision scar of the previous caesarean section. For this reason, we were able to carry out the research with very little contamination of fibrous tissue.

We add next sentence to method section:

“In the case of the secondary caesarean section, we performed a caesarean section with an incision cranial to the uterine incision scar of the previous caesarean section to minimize the presence of fibrous tissue”

**Comment:** Reviewer #2: Thank you for allowing me to review “Two oxytocin analogs, N-(p-fluorobenzyl) glycine and N-(3-hydroxypropyl) glycine, induce uterine contractions ex vivo in ways that differ from that of oxytocin » PPH is still an issue wordwide, and research is essential to find new safe and cost-effective utero tonic drug.

**Response**: Thank you very much for your review, interest to our research and good points.

**Comment:** Abstract :

Please reconsider the last sentence “they can be used as potent inducers of labor”

Concentrations of uterotonic are significantly lower in the induction of labor and this study was conduct to reduce the PPH. Thus you cannot conclude your abstract with this sentence.

**Response**: Thank you for your valuable comment. As you pointed out, we have deleted this sentence.

**Comment:** Introduction

« data obtained from laboring and non-laboring parturients have shown that carbetocin a long acting… »

Indeed, the carbetocin dont need an additionnal uterotonic but the PPH effect is not that remarkable compared to other uterotonic especially regarding the cost-effectiveness

**Response**: Thank you very much for your valuable comment. That’s another point, why we still should look for new cost-effective uterotonics.

**Comment:** Method

Line 136 “Women 136 with multiple pregnancies or medical conditions, such as diabetes, preeclampsia, or 137 obstetric cholestasis, were not included in this study »

Did you consider the BMI of patient?

**Response**: No, we did not take in account BMI in current study

**Comment:** Line 147 “Myometrial biopsies were excised 147 within 3 min of OT administration. »

Was it compromising that the human myometrial biopsie was realized 3 min after 5UI of OT?

**Response**: Thank you for good question. The clinical benefits of adding oxytocin to caesarean sections are clear and ethically unavoidable. After removal of the specimen, it is immediately immersed in PBS in the operating room, and we believe that the effects of remaining oxytocin are minimal.

**Comment:** Result

Line 211 “Effects of oxytocin analogs on contraction of human uterine muscles »

How do yo explain that carbetocin induced less contraction than OT?

**Response**: Considering pharmacodynamics, Carbetocin is partial agonist of Oxytocin receptors with efficacy (E_max_) lower than oxytocin (full agonist), this is was confirmed in previous studies on cell lines *in vitro* and confirmed here *ex vivo*. So, carbetocin can not activate as much receptors as oxytocin and unable produce same amount of contraction regardless of dosage. However, carbetocin have long-lasting activity, which beneficial in clinical context.

**Comment:** Discussion

Line 382 “Simultaneous use of FBOT 385 and OT displayed additivity, whereas identical effect was not observed with HPOT and 20 386 OT. »

The management of PPH often involves the use of a combination of uterotonic drog. Did you evaluate the combination: FBOT+ OT and HPOT+ OT and carbotacin

**Response**: Thank you for good point. We tested ex vivo interaction of FBOT+OT and HPOT+OT (Table 2), but did not test a lot of possible combinations such are combinations of Carbetocin+FBOT, Carbetocin+HPOT or FBOT+ OT and HPOT+ OT and carbotocin. While its is clinically important, there is limitation in our study. We address it in discussion and add next line:

“From a clinical point of view, it is important to test different possible combinations of uterotonics including a combination of FBOT or HPOT and Carbetocin, or a combination of more than 2 uterotonics. This issue should be addressed in future studies. “

**Comment:** Line 423 “However, in this 424 study, we tried to reduce these variations by carefully excising the marginal portion of 425 the incised uterus during the cesarean section. »

An other limit should be notice. The myometrial biopsies were excided in the transverse uterin incision, witch correspond to the uterine isthmus. This region is thinner and contains then less uterine muscle. Those results could be underestimated

**Response**: In our case myometrial biopsies were obtained from the upper margin of the incision made at the lower segment of the uterus.
